# The impacts of non-farming income on rural household energy choices: Empirical evidence from China

**DOI:** 10.3389/fpsyg.2022.1044362

**Published:** 2022-10-21

**Authors:** Gang Peng, Jie Zhang, Menghang Tang, Zhimin Duan

**Affiliations:** ^1^School of Statistics, Southwestern University of Finance and Economics, Chengdu, Sichuan Province, China; ^2^School of Statistics, Tianjin University of Finance and Economics, Tianjin, China

**Keywords:** non-farming income, rural household energy, energy upgrade, empirical evidence, social context

## Abstract

This study uses data from the China Family Panel Studies to analyze the possible impact of non-farming income on household energy choices. We use ordinary least squares and instrumental variable estimation methods to investigate the causal effect of non-farming income on household energy choices. We find that an increase in non-farming income assisted farmers in reducing their use of solid fuels in favor of clean energy. Our heterogeneity analysis, based on the average rural household income and geographical location of the village, shows that the energy upgrade effect of non-farming income is more obvious in high-income areas and suburbs closer to the county seat center. Further, we find that non-farming income has an impact on rural household energy choice mainly through the optimization of household energy-saving appliances and the enhancement of environmental awareness.

## Introduction

Using clean energy to protect the earth’s ecological environment is of great significance to all humanity. However, in China, there are many rural low-income families whose conditions are not conducive to using clean energy (Liao, 2019). In 2018, China’s rural traditional solid biomass energy for domestic use was about 130 million tons of standard coal, accounting for about half of the country’s total domestic energy. Rural households use firewood (44%) and coal (24%) as their main cooking and heating fuels (Chen J., 2019; Liao, 2019). Inefficient combustion of these solid fuels easily causes indoor pollution and has negative effects on the external environment. Currently, China is the second-largest polluter in the world, and rural energy consumption undoubtedly plays a key role in this (Guo and Wu, 2019). China aims to peak its carbon dioxide emissions by 2030 and achieve carbon neutrality by 2060. These emissions will continue to rise unless rural energy is transformed and upgraded. Following the experiences in global development, if the added value of the tertiary industry in a country or region is close to 50%, it should have the economic structural foundation for energy transformation (Leach, 1992). The added value of China’s tertiary industry reached 53.9% in 2019, suggesting that it may currently be appropriate to implement an energy transition. Consequently, it is of great significance to discuss how to promote the conversion of Chinese rural households from traditional to clean energy to reduce pollutant emissions in China and the global ecological environment.

The choice of rural household energy has long been a focal point of academic work. According to the energy ladder hypothesis, an increase in household income gradually shifts the choice of household energy away from traditional biomass fuels to clean energy (Leach, 1992). Compared with urban areas, China’s rural regions are characterized by relatively obvious features (Wei and Yu, 2022). Consequently, rural households are more reliant on traditional fuels, such as fuelwood and kerosene lamps—than urban households, and use relatively little clean energy, such as natural gas and electricity (Mottaleb et al., 2017). At the household level, poverty and low awareness of environmental issues have a significant impact on the energy choices of rural households (Su et al., 2016; Rahut et al., 2017; Saidi et al., 2017; Mao et al., 2018). At a regional level, the extent of economic development and geographical location are also key factors (Pachauri and Jiang, 2008). The extant research has, thus, deepened our understanding of rural household energy choices.

In recent years, with the advancement of China’s urbanization, increasingly more of the rural labor force chooses to venture out to engage in non-farming work. Rural families’ production and life decisions, including those on energy consumption, have undergone profound changes (Démurger and Fournier, 2007). However, only a few studies have investigated how non-farming income affects the energy choices of rural households. Using household survey data in Bhutan, Rahut et al. (2016) found that non-farming employment has a significantly positive impact on energy consumption such as natural gas and electricity. Although this study does not overcome the potential endogeneity problem, it nevertheless provides a profound insight into the effect of non-agriculture income on household energy choice. Two studies on China also found that non-agriculture employment promoted rural energy transition and upgrading, although only in non-poor areas (Shi et al., 2009; Ma et al., 2019). Compared to high-income households, low-income households often lack adequate access to clean energy and energy services, thus limiting their options for efficient energy (Gerarden et al., 2015; Gertler and Bennett, 2015). Moreover, because of the low efficiency of energy use, the economic activity time of low-income families is shortened, and the difficulty of improving clean energy inhibits the enhancement of their overall welfare (Alberini et al., 2018; Salameh et al., 2022)—there appears to be a vicious cycle between the inefficient use of energy and the low income of rural households. Moreover, current studies only discuss coal, electricity, and natural gas, while neglecting firewood and bottled liquefied natural gas, which are the most commonly used household energy sources in rural China.

The influence of non-agricultural employment and income on household energy choice is driven by: (i) the improvement of low-energy use household appliances; and (ii) the enhancement of environmental awareness. The relatively high cost of energy-saving appliances is the major obstacle to preventing the use of modern clean energy, whereas non-agricultural employment will lead to increased household income levels and significantly promote the upgrading of appliances for family use—abandoning traditional earthen stoves, using high-quality coal or gas stoves, and purchasing more electrical products (Shi et al., 2009; Zhang, 2016)—which assists farmers in switching to modern clean energy. Secondly, because farmers have lived in remote areas for extensive periods, they are relatively unaware and indifferent to the environmental protection policies issued by the government. Working in urban non-farming employment can effectively remediate the environmental awareness and values of rural residents (Zhou and Yang, 2009), thereby substantially changing their energy consumption habits (Dong and Xu, 2018) to that of modern clean energy. Therefore, income from non-farming employment has an important impact on the energy choices of rural households.

This study thus analyzes the impact of non-farming income on rural households’ energy choices and provides a reference for the government to promote the transformation of rural energy consumption structures. Specifically, we use CFPS data to examine changes in these energy structures based on empirical tests of non-farming income and energy choices, and the instrumental variable method to overcome the potential endogeneity problem. Further, by grouping rural households according to their *per capita* income level and regional location and analyzing their heterogeneity, we compare the effect of non-farming income on rural energy transition across high-income and low-income villages. Finally, we empirically test the ways the mechanisms of non-farming income influence energy choices from two perspectives: the improvement of energy appliances and the enhancement of environmental awareness.

This study makes three primary contributions to the existing literature. First, we provide new insights into the impact of non-farming income on household energy, by analyzing firewood and canned liquefied gas, which are more commonly used by Chinese rural households. Second, to understand possible regional differences in the impact intensity, heterogeneity was tested using *per capita* income levels and geographical location of villages, providing regional evidence for understanding the effect of energy choices. Third, we discuss in detail the channels and ways in which non-farming income influences the energy choices of rural households, providing important policy recommendations for the Chinese government to promote the transformation of the structure of energy consumption in rural areas.

## Empirical background

Since the beginning of the 21st century, China’s economy has shown sustained high growth with the government paying increasingly more attention to energy choices in rural areas and promoting the concept of environmental protection to rural households. In particular, biogas and electrical energy have been targeted by policies and legislation. Biogas project construction and home appliance subsidies into the countryside have become an important driving force to promote the transformation from traditional rural energy to clean energy. After nearly two decades of economic development and policy advancement, China’s rural energy transformation has likely made great progress. Therefore, we use the CFPS data from 2014, 2016, and 2018 to examine the empirical facts on energy utilization in rural China.

**Figure 1:**
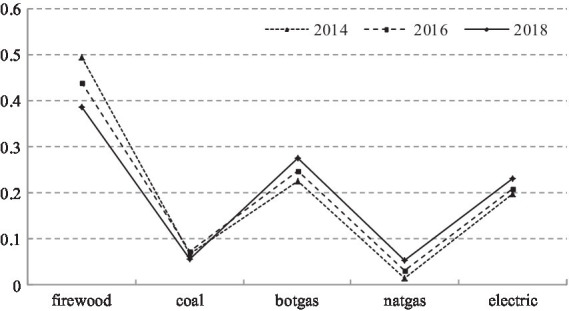
Changes in the structure of rural energy use.

Figure 1 indicates that China’s rural energy structure has significantly improved. The utilization of traditional non-clean energy represented by firewood and coal has decreased year on year, while the utilization of gas (bottled and natural) and electricity has increased significantly. Specifically, the proportion of households using firewood as their main energy source decreased from 49.6% in 2014 to 38.6% in 2018, while the proportion using coal decreased from 6.7% in 2014 to 5.5% in 2018. Conversely, the proportion of households using gas (bottled and natural) and electricity increased from 22.6%, 1.4%, and 19.7% to 27.6%, 5.2%, and 23.1% in 2014 and 2018, respectively. Moreover, firewood remains the primary source of household energy in rural areas, followed by bottled gas and electricity. The use of natural gas remains inadequate, mainly because most rural areas have not yet been piped (Qin et al., 2017; Liao, 2019).

The improvement in the mix of rural energy consumption is largely due to households amending their livelihood strategy. Before the 1990s, rural China was predominantly a small-scale peasant economy with rural households engaging in traditional agriculture and livestock farming for their livelihood, with their corresponding energy consumption dominated by non-clean sources such as firewood. Following the practice of the rural household contract system in the 1980s, labor demand gradually increased. In the 1990s, China’s township enterprises, especially those in coastal areas, had developed rapidly, with booming construction of all kinds in development zones. The rapid growth of the non-state sector exerted a strong demand for cheap rural labor. Since then, migrant work—in the pursuit of more abundant non-farming income—has become the livelihood choice of rural households in underdeveloped and impoverished areas in China to improve their living conditions. Accordingly, the energy choice of rural households in China has also changed significantly.

**Figure 2:**
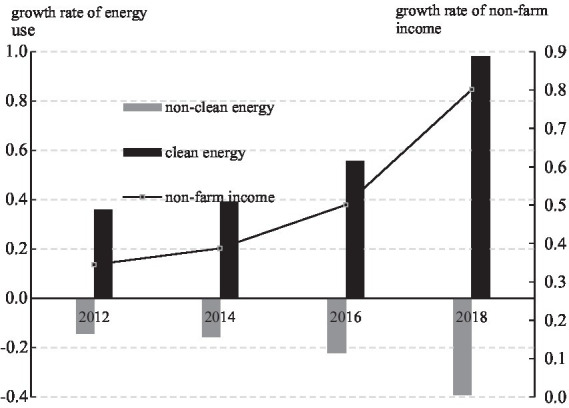
Trends in non-farming income and energy use (CFPS).

We describe this relationship by using CFPS data on household energy choices and non-farming income in 2012–2018. First, we categorize energy into clean (firewood and coal) and non-clean [gas (bottled and natural) and electricity] energy sources. The conversion is standardized using coal as a base to obtain the proportion of clean to non-clean energy. Second, we calculate the average non-farming income of all rural households with 2012 as the base period, using rural CPI to eliminate the impact of price factors. Finally, the growth rate of the utilization of the two types of energy and the growth rate of non-farming income are plotted in Figure 2, indicating that clean energy and non-farming income have a strong positive correlation, whereas the converse is indicated for non-clean energy. This is consistent with the energy ladder theory, which states that as income increases, residents who use non-clean energy will gradually switch to clean energy.

**Figure 3:**
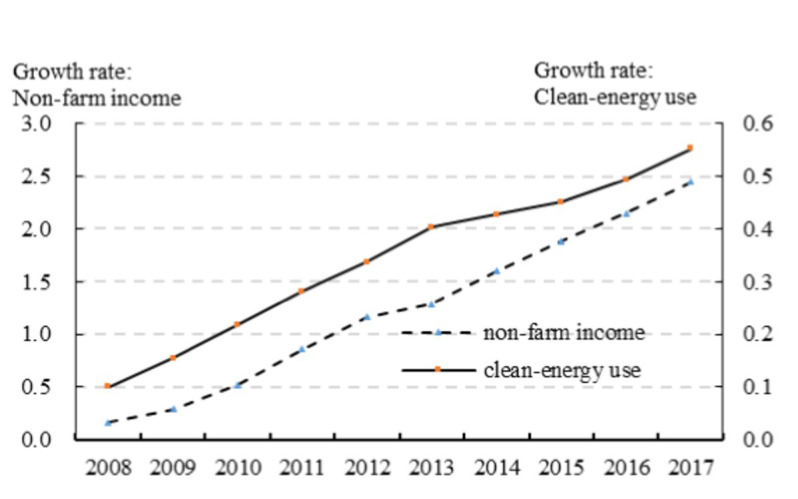
Trends in non-farming income and clean energy use (Yearbook).

To investigate the trend of synergistic change between non-farming income and household energy choice over a longer period, we use rural energy utilization data and wage income data from China Energy Statistical Yearbook and China Agricultural Statistical Yearbook for the 2009 to 2018 period to further describe typical facts of changes in wage income and household energy choices. It should be noted that the main energy used in daily life in the China Energy Statistical Yearbook only comprises coal, natural gas, and electricity. The three types of energy are divided into two groups: non-clean energy (coal) and clean energy (natural gas, electricity). As can be seen from Figure 3, clean energy utilization and non-farming income also have a strong trend of synergistic change—an increase in non-agricultural income increases the proportion of clean energy utilization—supporting the empirical evidence presented in Figure 2. However, empirical evidence does not necessarily imply a causal relationship between non-farming income and household energy use, and thus a more detailed investigation on the causal relationship, with more rigorous empirical methods, is required.

Moreover, Figure 2 shows that the growth rates (positive and negative) of clean non-clean energy utilization during 2016–2018 are significantly higher than in 2014–2016. This may be caused by the energy policy issued by the Chinese government in 2016 to pilot replacing coal with gas in some provinces, which was then intensively implemented on a nationwide scale from 2017 to 2018. It encouraged rural households to relinquish coal-based non-clean energy sources in favor of gas (bottled and natural) and electricity-based energy by offering financial subsidies and economic incentives. This greatly influenced the energy utilization of rural households and strengthened the substitution of clean with non-clean energy sources (Xu et al., 2019; Xie et al., 2020). To distinguish between our empirical findings and the policy of replacing coal with gas, we conducted appropriate robustness tests.

## Data and methods

### Data

The data in this study are from the China Family Panel Studies (CFPS). The survey covers 162 counties in 25 provinces, municipalities, and autonomous regions. Since the official launch of the survey in 2010, it has been conducted every 2 years to collect data at the individual, household, and community levels to reflect China’s socioeconomic, demographic, educational, and environmental changes, providing a data foundation for academic research and public policy analysis. Due to substantial missing data on non-farming income in 2010 and 2012, we select rural households in 2014, 2016, and 2018 as the final samples. We process the household data as follows: first, the unrecognized samples are deleted if any variables such as community code, family code, individual code, urban and rural code are missing; second, any households headed by people under the age of 16 are excluded; third, to avoid the impact of outliers, households in the top and bottom 1% of non-farming income are excluded; and fourth, samples with other missing variables involved in the empirical model are deleted. These results in a sample size of 19,453.

### Empirical model

The explained variable in this study is whether a household chooses a particular energy type as the main source of energy—a dichotomous variable. The simple ordinary least squares (OLS) method is used to initially estimate the impacts of non-farming income on rural household energy choices. Thus, the Probit model is adopted to estimate it using the following benchmark model:


(1) $$p\left(rer_{ij}=1|\ln\;\;n\;\;oninc_{ij},X_{ij}\right)=\beta \;\;\ln\;\;n\;\;oninc_{ij}+\lambda X_{ij}+\delta_{j}+\varepsilon_{ij}$$

where $rer_{ij}$ is the choice of energy source for household i in village j . If this energy is used as the main source of household energy, its value is 1; otherwise, it is 0. Because this study mainly focuses on firewood, coal, gas (bottled and natural), and electricity, each type of domestic energy will be a regression in the subsequent regression. *ln n oninc_ij_* is the logarithm of non-farming income of household i in village j . Considering that agricultural income is not based on wages, whereas wage income is dominated by non-farming employment, this study takes the wage income of households as the measure of their non-farming income. Taking 2014 as the base period, the non-agricultural income is deflated by the cumulative annual rural consumer price index.

To better mitigate the estimation bias caused by the omitted variables, the relevant characteristics of household head and family are also controlled in this study. Specifically, different families have different working ability and living habits, more attention should be paid to the physical properties of energy, such as the weight or cleanliness of the energy (Han et al., 2014; Rahut et al., 2016, 2017). Moreover, non-farming employment can increase the environmental awareness of rural households, but this depends on education levels (Dong and Xu, 2018). Moreover, if the household has a many young or elderly people, the feasibility of using clean energy may be affected by the difficulty in operating the appliance, so the child-and the elderly-dependency ratios of the household are also controlled. Therefore, we included age, gender, health status, and education level of the householder in the model as control variables.

### Instrumental variables

The long-term existence of the urban–rural dual system in China has led to a stark contrast in economic development between rural and urban areas. In China’s rural areas, many families have chosen non-farming work to increase their incomes. These households have an advantage over non-migrant workers in terms of education, physical fitness, and skill (Bai and Duan, 2013) and other significant, but unobservable characteristics (Babatunde and Qaim, 2010; Burgess et al., 2017). Moreover, there is a reverse causality between non-farming employment and household energy use. Low-income households lack the economic ability to obtain clean energy, while extended acquisition times and inefficient traditional non-clean energy limits the time available by households engaged in non-farming employment (Mekonnen et al., 2017; Yang et al., 2020). Although the fixed effects of household head, household, and village characteristics are controlled, the endogeneity problem in the model [see equation (1)] persists, making any estimation results in this study biased and inconsistent. To obtain a more accurate causal association, it is still necessary to find instrumental variables (IVs) that are related to rural households’ *per capita* non-farming income but unrelated to their energy choices.

Several studies show that the distance of migrant workers from their homes has a significant positive impact on the wage level of the labor force—a greater distance results in larger income (Ning, 2012; Liu, 2015; Wang and Zhou, 2015)—as do working hours, with longer working hours implying higher labor intensity and labor costs. This should be compensated by higher wages according to the principle of exchange of equivalence. Although these have an important impact on non-farming income, there is no correlation between them and rural households’ energy choices, and thus, they serve as the IVs of rural households’ non-farming income to solve the potential endogeneity problem. Detailed explanations of the above variables and simple descriptive statistical results are shown in Table 1.

**Table 1:** Statistical description of main variables (*n* = 19,453).

Variable	Name	Specific explanation	Mean	SD	Max value	Min value
Firewood	firewood	Whether firewood i the main source of energy (if yes, the value is 1; otherwise, it is 0)	0.44	0.49	1	0
Coal	coal	Whether coal is the main source of energy (if yes, the value is 1; otherwise, it is 0)	0.06	0.24	1	0
Bottled gas	botgas	Whether bottled gas is the main source of energy (if yes, the value is 1; otherwise, it is 0)	0.24	0.43	1	0
Natural gas	natgas	Whether natural gas is the main source of energy (if yes, the value is 1; otherwise, it is 0)	0.03	0.17	1	0
Electricity	electric	Whether electricity is the main source of energy (if yes, the value is 1; otherwise, it is 0)	0.21	0.41	1	0
Non-farming income	lnnoninc	Logarithm of wage income	7.64	4.23	8.85	1
The gender of householders	gender	Whether the householder is a woman (if yes, the value is 1; otherwise, it is 0)	0.44	0.50	1	0
The age of householder	age	The age of householders	50.46	14.06	94	16
The education level of householders	educ	The education years of householders	6.69	3.36	19	0
The health of householder	health	1-unhealthy, 5-very healthy	2.90	1.28	5	1
Child dependency ratio	child_r	The proportion of persons under the age of 16 in a household	0.14	0.17	1	0
Elderly dependency ratio	old_r	The proportion of persons over 65 years of age in a household	0.13	0.26	1	0
Migrant work distance (kilometer)	distance	The average distance of all migrant workers in a household (1-working in the village, 2-working across the village, 3-working across the country, 4-working across the city, 5-working across the province.)	1.82	1.10	5	1
Working hours (per person per week)	time	The average working hours for all migrant workers in a household	47.15	21.92	84	42

## Estimation results

### Baseline regression

Based on the model, a probit estimation is conducted on the impact of non-farming income on household energy choice. We report the estimated results of firewood, coal, gas (bottled and natural), and electricity in Panels A–E, respectively. Column one is the estimated results without adding any control variables, column two controls for the relevant characteristics of householders, and column three is further controlled at the family level. Moreover, for all regression results, households’ village and year fixed-effects are controlled. The complete estimated results of control variables are shown in the Appendix.

As presented in Table 2, an increase in rural households’ non-farming income has a significant negative impact on the use of traditional energy sources such as firewood and coal, remaining stable after successively adding householder characteristics and family characteristics. Specifically, we found that the estimated marginal effect of non-farming income on firewood was-0.028, which means a 1% increase in non-farming income would reduce the likelihood of households using firewood as their primary energy source by 2.8 percentage points, equivalent to 6.4% of the firewood used. Moreover, the increase in non-farming income has a significant negative impact on the use of coal—when other conditions remain unchanged, the probability of households using coal as the main energy source decreases by 0.7 percentage points for every 1% increase in non-farming income, to 11.7% of the coal used. Moreover, an increase in non-farming income is conducive to the use of gas (bottled and natural) and electricity, although the use of electricity is not significant after the inclusion of householder and family characteristics. Among them, if other conditions remain unchanged, a 1% increase in household non-farming income results in a 3.1 and 2.6 percentage point increase in the probability of using bottled and natural gas as the main energy source, respectively. This indicates that with an increase in non-farming income, clean energy has a significant substitution effect on traditional energy among the main energy sources used by rural households, which is consistent with the energy ladder theory whereby an increase in household income tends to lead to an increase in clean energy use, thus promoting energy use transformation in rural areas.

**Table 2:** Baseline regression results.

	Column one	Column two	Column three
**Panel A Firewood (** firewood **)**			
*ln n oninc*	−0.014^***^ (0.001)	−0.007^***^ (0.001)	−0.009^***^ (0.0008)
Householder characteristics		√	√
Family characteristics			√
Village fixed effect	√	√	√
Year fixed effect	√	√	√
Intercept	0.085 ^***^ (0.017)	0.681^***^ (0.061)	0.602^***^ (0.064)
$R^{2}$	0.013	0.051	0.051
Observations	19,451	19,451	19,451
**Panel B Coal (** coal **)**			
*ln n oninc*	−0.001^***^ (0.0004)	−0.001^***^ (0.0004)	−0.0008^*^ (0.00042)
Householder characteristics		√	√
Family characteristics			√
Village fixed effect	Yes	Yes	Yes
Year fixed effect	Yes	Yes	Yes
Intercept	−1.458^***^ (0.025)	−1.599^***^ (0.092)	−1.641^***^ (0.097)
$R^{2}$	0.001	0.002	0.004
Observations	19,451	19,451	19,451
**Panel C Bottled gas (** botgas **)**			
*ln n oninc*	0.010^***^ (0.0007)	0.008^***^ (0.0007)	0.008^***^ (0.0007)
Householder characteristics		√	√
Family characteristics			√
Village fixed effect	Yes	Yes	Yes
Year fixed effect	Yes	Yes	Yes
Intercept	−0.940^***^ (0.020)	−1.489^***^ (0.068)	−1.459^***^ (0.070)
$R^{2}$	0.011	0.026	0.026
Observations	19,451	19,451	19,451
Panel D Natural gas ( natgas )			
*ln n oninc*	0.003^***^ (0.0003)	0.002^***^ (0.0003)	0.002^***^ (0.0003)
Householder characteristics		√	√
Family characteristics			√
Village fixed effect	Yes	Yes	Yes
Year fixed effect	Yes	Yes	Yes
Intercept	−2.183^***^ (0.042)	−2.414^***^ (0.127)	−2.256^***^ (0.132)
$R^{2}$	0.016	0.064	0.068
Observations	19,451	19,451	19,451
**Panel E Electricity (** electric **)**			
*ln n oninc*	0.002^***^ (0.0006)	0.001 (0.001)	0.001 (0.001)
Householder characteristics		√	√
Family characteristics			√
Village fixed effect	Yes	Yes	Yes
Year fixed effect	Yes	Yes	Yes
Intercept	−0.862^***^ (0.019)	−1.162^***^ (0.068)	−1.187^***^ (0.071)
$R^{2}$	0.0004	0.004	0.005
Observations	19,451	19,451	19,451

() is the clustering robust standard error at the village level, ^*^statistical significance at 10% level, ^**^statistical significance at 5% level, ^***^statistical significance at 1% level.

When comparing the marginal effects of different energy estimates, we initially find that the effect of non-farming income on the probability of firewood use is stronger than coal, providing further support for the empirical evidence of the energy ladder theory. According to this theory, the energy use of rural households has three stages: inferior energy (firewood and dung), low-quality energy (kerosene and coal), and high-quality energy (bottled and natural gas, electricity). Low-income households tend to use inferior energy, and as incomes rise, priority shifts to the second stage, with correspondingly greater reliance on kerosene and coal as primary sources of energy. Thus, although coal is a conventional energy source, the reduction in its use is less than that of firewood. Secondly, the marginal lift effect of non-farming income on bottled gas is more obvious than that of natural gas, arising from the associated costs of using each. Typically, the price of bottled gas is lower than natural gas, hence rural households are more inclined to use it as their income rises. Moreover, the use of natural gas relies on the relatively expensive installation of specialized gas pipelines by local governments, while the use of bottled gas is more convenient with very low installation costs.

According to the estimation results of the control variables, the gender, age, education and health level of the householder, and the number of young and elderly members in a family have a significant impact on the energy decision (Rahut et al., 2016; Mottaleb et al., 2017; Dong and Xu, 2018). Specifically, female householders have a significantly negative impact on the use of firewood, and a significantly positive impact on gas (bottled and natural) and electricity, indicating that they are more inclined to use clean energy. The age of householder has a significant negative effect on natural gas and a significant positive effect on electricity and firewood. The education level of the householder has a significant negative impact on the use of firewood, and a significant positive impact on the use of gas (bottled and natural) and electricity, indicating that householders with higher education levels are more inclined to choose clean energy. The level of health of householders has a significant negative effect on firewood, and a significant positive effect on coal, natural gas, and electricity. The number of young family members has a significantly positive effect on the use of firewood and coal and a significantly negative effect on the use of natural gas. The elderly populations have a significant negative effect on the use of firewood and electricity, and a significant positive effect on the use of coal and bottled gas.

### IV estimation results

Although the OLS estimation can initially reflect the relationship between non-farming income and rural household energy choices, this does not explain its causal relationship due to potential endogenous effects. To identify the causal relationship between off-farm income and household energy choice more accurately, it is necessary to overcome the endogeneity problems arising from selection bias and the omission of variables. We choose migrant work distance and working hours as the IVs, presenting their first stage estimation results in Table 3—Column one (no control variables), column two (relevant characteristics of householders), and column three (controlled at the family level).

**Table 3:** IV estimation results of the first stage.

	Column one	Column two	Column three
distance	0.912^***^ (0.030)	0.702^***^ (0.030)	0.662^***^ (0.029)
time	0.017^***^ (0.001)	0.012^***^ (0.001)	0.008^***^ (0.001)
gender		0.492^***^ (0.065)	0.520^***^ (0.063)
age		−0.065^***^ (0.003)	−0.026^***^ (0.003)
educ		0.136^***^ (0.010)	0.130^***^ (0.010)
health		−0.023 (0.025)	0.006 (0.025)
child_r			−0.013 (0.186)
old_r			−4.393^***^ (0.134)
Village fixed effect	Yes	Yes	Yes
Year fixed effect	Yes	Yes	Yes
Intercept	5.797^***^ (0.079)	8.440^***^ (0.216)	7.217^***^ (0.222)
$R^{2}$	0.057	0.117	0.164
Observations	19,445	19,445	19,445

() is the clustering robust standard error at the village level, ^*^statistical significance at 10% level, ^**^statistical significance at 5% level, ^***^statistical significance at 1% level.

As evidenced in Table 3 and according to our expectation, the average distance and working hours of migrant workers have a significant impact on the non-farming income of rural households, with or without the control variables. The average distance has a stronger positive impact than working hours, which can provide insight into the huge scale of labor migration in China from the relatively underdeveloped central and western regions to the eastern coastal cities.

The value of the Kleibergen-Paap rk Wald F statistic is 140.39, greater than the critical value (19.93) of the weak instrumental variable hypothesis that was rejected at the 10% significance level, indicating that there is no weak instrumental variable problem. Moreover, according to the Durbin–Wu–Hausman test results, the corresponding *p*-values of firewood, coal, bottled gas, natural gas, and electricity are 0.000, 0.001, 0.001, 0.000, and 0.059 respectively, strictly rejecting the hypothesis of exogenous household non-farming income. For the exogeneity of IVs, the *p*-values of the Sargan test for the five types of energy are all >0.05, which cannot reject the null hypothesis that IVs are not related to the error term of the regression equation—IVs satisfy the erogenicity constraint. Therefore, the IVs selected in this study are appropriate and effective, and the estimation results obtained in the second stage are shown in Table 4.

**Table 4:** IV estimation results of the second stage.

	Column one	Column two	Column three
**Panel A Firewood (** firewood **)**			
*ln n oninc*	−0.041^***^ (0.003)	−0.033^***^ (0.004)	−0.037^***^ (0.005)
Householder characteristics		√	√
Family characteristics			√
Village fixed effect	√	√	√
Year fixed effect	√	√	√
Intercept	0.729^***^ (0.024)	1.002^***^ (0.049)	0.964^***^ (0.048)
Durbin–Wu–Hausman *p*-value	0.000	0.000	0.000
Sargan test *p*-value	0.022	0.121	0.161
Observations	19,445	19,445	19,445
**Panel B Coal (** coal **)**			
*ln n oninc*	−0.002 (0.002)	−0.003 (0.002)	−0.002 (0.002)
Householder characteristics		√	√
Family characteristics			√
Village fixed effect	√	√	√
Year fixed effect	√	√	√
Intercept	0.081^***^ (0.012)	0.069^***^ (0.024)	0.062^***^ (0.024)
Durbin–Wu–Hausman *p*-value	0.001	0.001	0.001
Sargan test *p*-value	0.448	0.476	0.526
Observations	19,445	19,445	19,445
**Panel C Bottled gas (** botgas **)**			
*ln n oninc*	0.012^***^ (0.003)	0.007^**^ (0.003)	0.009^**^ (0.004)
Householder characteristics		√	√
Family characteristics			√
Village fixed effect	√	√	√
Year fixed effect	√	√	√
Intercept	0.157^***^ (0.021)	0.017 (0.042)	0.017 (0.041)
Durbin–Wu–Hausman *p*-value	0.000	0.001	0.001
Sargan test *p*-value	0.420	0.715	0.867
Observations	19,445	19,445	19,445
**Panel D Natural gas (** natgas **)**			
*ln n oninc*	0.016^***^ (0.001)	0.016^***^ (0.002)	0.018^***^ (0.002)
Householder characteristics		√	√
Family characteristics			√
Village fixed effect	√	√	√
Year fixed effect	√	√	√
Intercept	−0.084^***^ (0.009)	−0.157^***^ (0.018)	−0.132^***^ (0.018)
Durbin–Wu–Hausman *p*-value	0.000	0.002	0.000
Sargan test *p*-value	0.129	0.664	0.669
Observations	19,445	19,445	19,445
**Panel E Electricity (** electric **)**			
*ln n oninc*	0.008^***^ (0.003)	0.007^**^ (0.003)	0.008^**^ (0.004)
Householder characteristics		√	√
Family characteristics			√
Village fixed effect	√	√	√
Year fixed effect	√	√	√
Intercept	0.148^***^ (0.020)	0.043 (0.040)	0.039 (0.039)
Durbin–Wu–Hausman *p*-value	0.012	0.057	0.059
Sargan test *p*-value	0.899	0.857	0.862
Observations	19,445	19,445	19,445

() is the clustering robust standard error at the village level, ^*^statistical significance at 10% level, ^**^statistical significance at 5% level, ^***^statistical significance at 1% level.

The IV estimation results show that the impacts of non-farming income on the probability of using firewood and gas (bottled and natural) is consistent with the benchmark estimation results in direction and significance, although greater because endogeneity causes the benchmark estimate to tend toward zero. Specifically, for every 1% increase in non-farming income, the probability of using firewood as the main energy of rural households on average decreases by 3.7%, while the probability of using gas (bottled and natural) increases by 0.9% and 1.8%, respectively. It should be noted that after overcoming the endogeneity problem in the model, the impact of non-farming income on the probability of coal and electricity use is significantly different from the benchmark estimate. Specifically, we find that non-farming income has a significant positive effect on the probability of electricity use, possibly because the increase in non-farming income makes rural households more likely to access the internet (Li, 2011), which makes it more convenient for families to use online shopping (Kulshreshtha et al., 2017) to purchase domestic appliances (Ma et al., 2019). This would encourage rural household to switch to electricity. Moreover, we found that non-farming income no longer had a significant effect on coal use. This is different from the research conclusions of Ma et al. (2019), and Shi et al. (2009), who found that increases in non-farming income had a negative impact on household spending on coal. We argue that as coal is mainly used for winter heating in rural households in China, especially in the north (Xiao et al., 2017), coal use by farmers follows a rigid demand with little correlation with non-farming income. In recent years, many studies have analyzed the impact of China’s heating policy on environmental pollution due to the use of coal as the main energy source, indicating that in southern China where the heating policy is not applicable, coal is used less and the impact on pollution is lighter (Chen et al., 2013; Ebenstein et al., 2015).

### Robustness test

To verify the robustness of the above-estimated results, four aspects are tested.

First, the Probit model assumes that the random error term obeys the normal distribution. This study uses the Logit model to re-estimate and overcome the adverse effects created by this strong assumption as shown in Table 5. The results show that the coefficient sign and significance of the estimation results of the Logit model are consistent with the estimation results of the Probit model.

**Table 5:** Robustness Logit test regression results.

	Firewood ( firewood )	Coal ( coal )	Bottled gas ( botgas )	Natural gas ( natgas )	Electricity ( electric )
*ln n oninc*	−0.106^***^ (0.012)	−0.020 (0.021)	0.031^**^ (0.014)	0.159^***^ (0.014)	0.027^**^ (0.014)
Householder characteristics	√	√	√	√	√
Family characteristics	√	√	√	√	√
Village fixed effect	√	√	√	√	√
Year fixed effect	√	√	√	√	√
Intercept	1.191^***^ (0.106)	−1.536^***^ (0.190)	−1.469^***^ (0.129)	−3.064^***^ (0.125)	−1.382^***^ (0.124)
Durbin–Wu–Hausman *p*-value	0.100	0.367	0.032	0.000	0.046
Sargan test *p*-value	0.254	0.479	0.565	0.145	0.649
Observations	19,445	19,445	19,445	19,445	19,445

() is the clustering robust standard error at the village level, ^*^statistical significance at 10% level, ^**^statistical significance at 5% level, ^***^statistical significance at 1% level.

Second, compared to the total non-farming income, the *per capita* non-farming income may reflect an improvement of living standards caused by an increase in the non-farming income of households, resulting in a more direct impact on the choice of household energy. Therefore, this study further adjusts the total non-farming income by household size and re-estimates the *per capita* non-farming income as the core explanatory variable as presented in Table 6; when *per capita* non-agricultural income was taken as the key explanatory variable, the results are similar to those in Table 4.

**Table 6:** Robustness tests: estimated by *per capita* non-farming income.

	Firewood ( firewood )	Coal ( coal )	Bottled gas ( botgas )	Natural gas ( natgas )	Electricity ( electric )
*ln n oninc*	−0.039^***^ (0.005)	−0.002 (0.002)	0.009^**^ (0.004)	0.019^***^ (0.002)	0.008^**^ (0.004)
Householder characteristics	√	√	√	√	√
Family characteristics	√	√	√	√	√
Village fixed effect	√	√	√	√	√
Year fixed effect	√	√	√	√	√
Intercept	0.946^***^ (0.045)	0.0613^***^ (0.023)	0.0207 (0.039)	−0.123^***^ (0.017)	0.0424 (0.038)
Durbin–Wu–Hausman *p*-value	0.000	0.216	0.000	0.000	0.010
Sargan test *p*-value	0.339	0.549	0.746	0.141	0.753
Observations	19,445	19,445	19,445	19,445	19,445

() is the clustering robust standard error at the village level, ^*^statistical significance at 10% level, ^**^statistical significance at 5% level, ^***^statistical significance at 1% level.

Third, in 2016 the Chinese government implemented the policy of replacing coal with gas throughout the country, which aims to encourage the use of natural gas as the energy source for winter heating while reducing the use of coal. Simultaneously, due to the spillover effect of the policy, the use of firewood by rural households was also restrained. The implementation of this policy may confuse the estimates in this study—we expect the negative impact of non-farming income on the probability of firewood and coal use and the positive impact on the probability of natural gas use to be *weaker* in the period before the implementation of the policy than that after. To verify this, we limited the sample period to 2014 for re-estimation, and the results are shown in Table 7—in line with our expectations.

**Table 7:** Robustness test: 2014 sample estimated results.

	Firewood ( firewood )	Coal ( coal )	Bottled gas ( botgas )	Natural gas ( natgas )	Electricity ( electric )
*ln n oninc*	−0.031^***^ (0.011)	0.003 (0.004)	0.029^**^ (0.014)	0.003 (0.003)	0.006^**^ (0.003)
Householder characteristics	√	√	√	√	√
Family characteristics	√	√	√	√	√
Village fixed effect	√	√	√	√	√
Year fixed effect	√	√	√	√	√
Intercept	0.999^***^ (0.098)	−0.015 (0.049)	−0.212^***^ (0.082)	−0.017 (0.023)	0.103 (0.078)
Durbin–Wu–Hausman *p*-value	0.003	0.624	0.010	0.196	0.015
Sargan test *p*-value	0.167	0.446	0.493	0.345	0.594
Observations	6,498	6,498	6,498	6,498	6,498

() is the clustering robust standard error at the village level, ^*^statistical significance at 10% level, ^**^statistical significance at 5% level, ^***^statistical significance at 1% level.

Finally, because wage income is taken as the replacement of family non-farming income, other wage income may be earned by labor in agricultural production for other households. Accordingly, we substitute the wage income obtained by rural households from the survey as the variable and re-estimate, as presented in Table 8—confirming the previously estimated results.

**Table 8:** Robustness test by wage income.

	Firewood ( firewood )	Coal ( coal )	Bottled gas ( botgas )	Natural gas ( natgas )	Electricity ( electric )
*ln w age*	−0.040^***^ (0.008)	0.004 (0.004)	0.002^***^ (0.0006)	0.012^***^ (0.002)	0.009 (0.006)
Householder characteristics	√	√	√	√	√
Family characteristics	√	√	√	√	√
Village fixed effect	√	√	√	√	√
Year fixed effect	√	√	√	√	√
Intercept	0.947^***^ (0.065)	0.004 (0.032)	0.056 (0.052)	−0.067^***^ (0.020)	0.027 (0.050)
Durbin–Wu–Hausman *p*-value	0.010	0.415	0.049	0.000	0.036
Sargan test *p*-value	0.062	0.564	0.159	0.391	0.506
Observations	12,861	12,861	12,861	12,861	12,861

() is the clustering robust standard error at the village level, ^*^statistical significance at 10% level, ^**^statistical significance at 5% level, ^***^statistical significance at 1% level.

## Further analyses

### Heterogeneity analysis

The impact of non-farming income on rural households’ energy choices may vary with geographical location and economic development level. In the Chinese context, understanding the heterogeneity of these impacts will help formulate precise and supportive measures to smoothly achieve China’s energy transformation and upgrading goals, warranting a detailed discussion of the heterogeneity at the village level.

#### The difference in village economic development level

In China’s rural areas, higher levels of economic development, with greater urbanization, results in more frequent communication with the city; thus, people are more susceptible to the influence of urban living habits and inclined to choose clean energy. Moreover, rural areas with better economic development are likely to take the lead in installing clean energy equipment, such as natural gas pipelines. Meanwhile, differences in the price of energy facilities also reinforce the impact of economic power on households’ energy choices, with households in high-income areas having more money to afford the cost of installing clean energy facilities. In traditional Chinese rural settlements, individuals with strong economic acquisition ability are more likely to become the model for other individuals, and their wealth, experience, and daily life behaviors will also have a demonstrable effect on others. This effect is more pronounced in villages with greater economic development, and therefore, we expect the energy upgrade effect of non-farming income to be more obvious in these villages.

Due to data limitations, however, we cannot establish the index value of the economic development level of each village. Considering the strong correlation between the level of economic development and the income of rural households, this study uses the total household income data in the same village to calculate the average income and uses this as the measure of the level of villages’ economic development. Using the median income level, the households are divided into two categories: households in high-income or low-income villages. The heterogeneity of non-farming income on energy choices is analyzed and the results are presented in Table 9.

**Table 9:** Heterogeneity test: Estimated by income level.

	Firewood ( firewood )	Coal ( coal )	Bottled gas ( botgas )	Natural gas ( natgas )	Electricity ( electric )
**Panel A: Lower-income villages**					
*ln n oninc*	−0.036^***^ (0.007)	−0.005 (0.004)	0.004 (0.005)	0.013^***^ (0.002)	0.017^***^ (0.006)
Householder characteristics	√	√	√	√	√
Family characteristics	√	√	√	√	√
Village fixed effect	√	√	√	√	√
Year fixed effect	√	√	√	√	√
Intercept	1.090^***^ (0.064)	0.108^***^ (0.034)	−0.011 (0.048)	−0.103^***^ (0.020)	−0.104^*^ (0.053)
Durbin–Wu–Hausman *p*-value	0.000	0.215	0.598	0.000	0.003
Sargan test *p*-value	0.435	0.005	0.000	0.289	0.317
Observations	9,125	9,125	9,125	9,125	9,125
**Panel B: Upper-income villages**					
*ln n oninc*	−0.048^***^ (0.007)	0.002 (0.003)	0.021^***^ (0.007)	0.018^***^ (0.003)	0.005 (0.006)
Householder characteristics	√	√	√	√	√
Family characteristics	√	√	√	√	√
Village fixed effect	√	√	√	√	√
Year fixed effect	√	√	√	√	√
Intercept	0.940^***^ (0.076)	−0.012 (0.033)	−0.054 (0.068)	−0.117^***^ (0.028)	0.187^***^ (0.063)
Durbin–Wu–Hausman *p*-value	0.000	0.317	0.226	0.000	0.169
Sargan test *p*-value	0.182	0.537	0.208	0.106	0.407
Observations	9,695	9,695	9,695	9,695	9,695

() is the clustering robust standard error at the village level, ^*^statistical significance at 10% level, ^**^statistical significance at 5% level, ^***^statistical significance at 1% level.

The results show that the energy upgrade effect of non-farming income varies with the economic development level of the villages. Specifically, in high-income villages, the negative marginal effect of non-farming income on firewood is higher than that of low-income villages, and the positive marginal effect on gas (bottled and natural) is stronger. However, it is noteworthy that non-farming income has no effect on the probability of electricity use in high-income villages but is significantly positive in low-income villages —the use of electricity in high-income villages has long been widespread. In general, in the high-income villages, the role of non-farming income in optimizing the transformation of energy choice is more obvious, consistent with our expectations.

#### Differences in the geographical location of villages

In recent years, the Chinese government has introduced a series of policies to optimize energy use in rural areas. Some rural areas are closer to urban areas, thus the implementation of the policy is stronger, while some areas cannot fully implement the policy due to excessive distance. Moreover, more remote rural households are farther away from markets, making it more expensive for them to obtain clean energy. Therefore, we posit that the energy upgrade effect of non-farming income should be significantly different in rural areas with different geographical locations. To investigate the impact of this heterogeneity, we classify the geographical location of the village according to its distance from the center of the county seat. Specifically, according to Wu (1997), if the distance is within 50 km, the settlement is defined as a suburban village, otherwise, it is defined as an exurb village. The regression results are shown in Table 10.

**Table 10:** Heterogeneity test: Estimated by geographical location.

	Firewood ( firewood )	Coal ( coal )	Bottled gas ( botgas )	Natural gas ( natgas )	Electricity ( electric )
**Panel A: Exurbs villages**					
*ln n oninc*	−0.043^***^ (0.007)	−0.0004 (0.003)	0.012^**^ (0.005)	0.012^***^ (0.002)	0.014^***^ (0.005)
Householder characteristics	√	√	√	√	√
Family characteristics	√	√	√	√	√
Village fixed effect	√	√	√	√	√
Year fixed effect	√	√	√	√	√
Intercept	1.052^***^ (0.066)	0.049 (0.031)	−0.045 (0.049)	0.071^***^ (0.018)	0.0039 (0.049)
Durbin–Wu–Hausman *p*-value	0.000	0.445	0.019	0.000	0.012
Sargan test *p*-value	0.054	0.641	0.122	0.682	0.569
Observations	9,531	9,531	9,531	9,531	9,531
**Panel B: Suburbs villages**					
*ln n oninc*	−0.015^**^ (0.007)	−0.002 (0.004)	−0.008 (0.007)	0.019^***^ (0.003)	0.004 (0.007)
Householder characteristics	√	√	√	√	√
Family characteristics	√	√	√	√	√
Village fixed effect	√	√	√	√	√
Year fixed effect	√	√	√	√	√
Intercept	0.633^***^ (0.071)	0.044 (0.036)	0.222^***^ (0.074)	−0.117^**^ (0.035)	0.119^*^ (0.069)
Durbin–Wu–Hausman *p*-value	0.030	0.472	0.007	0.000	0.039
Sargan test *p*-value	0.056	0.105	0.147	0.057	0.671
Observations	8,284	8,284	8,284	8,284	8,284

() is the clustering robust standard error at the village level, ^*^statistical significance at 10% level, ^**^statistical significance at 5% level, ^***^statistical significance at 1% level.

The results show that the negative influence of non-farming income on the probability of using firewood in the exurb villages is significantly stronger than that of farmers in suburban villages, possibly due to the widespread use of firewood in the exurb villages. Since firewood mainly comes from straws and the cultivated area in the exurbs is more extensive than that in the suburbs, firewood is relatively more abundant and more convenient to obtain. According to the energy ladder theory, as non-farming income increases, the decreasing effect on the probability of rural households in the exurbs using firewood is stronger. Moreover, when the non-farming income of rural households increases, the increasing effect of using natural gas as the main energy source is significantly stronger due to the intensity of policy implementation in rural areas across different geographical locations.

It is also noteworthy that in suburban villages, non-farming income no longer has a significant effect on the use of bottled gas and electricity. This may result from the radiation influence of the city on the suburban villages, which weakens the positive effect of non-farming income on the use of bottled gas and electricity. The impact has two aspects. First, urban lifestyles, and the central city in particular, directly affects the decision-making on energy choice through exerting a strong radiating and driving effect on suburban villages (Zhou and Yang, 2009), and weakens the impacts of non-farming income on these decisions. Second, urbanization will lead to changes in households’ consumption habits and ways of using energy (Chen M., 2019). Greater urbanization creates a higher starting point of energy consumption and clean energy, such as bottled gas and electric energy, thus occupies a greater proportion of the energy structure of rural households. This compresses the space for further optimization and transformation of the rural energy structure and greatly weakens the positive effect on the use of bottled gas and electricity until it is no longer significant.

### Mechanism analysis

Because the increase in non-farming income has a positive impact on the energy upgrade of rural households, it is necessary to explore how this impact is generated. Just as consumers’ demand for goods depends on their willingness and ability to buy, rural households’ energy upgrades also depend on their willingness and ability to use clean energy. The willingness of rural households to use clean energy is mainly determined by their environmental awareness, while their ability is determined by the effectiveness of their energy-efficient appliances. We analyze this mechanism from two perspectives: (i) whether non-farming income has an impact on rural households’ environmental awareness; and (ii) the use of energy-efficient appliances.

#### Impact of non-farming income on improvements In energy appliances

This study reflects the development of energy provision to rural households by using the rating of the investigators, ranging from 1 (poor energy-efficient appliances) to 7 (optimal energy-efficient appliances), with the estimated results are shown in Table 11. Non-farming income has a significantly positive effect on the household energy facility score—it can positively influence the choice of energy by improving household energy provision—by replacing traditional earth stoves with better coal and gas stoves, and the purchase of other electrical household appliances (Shi et al., 2009; Zhang, 2016). This will help farmers gradually relinquish traditional solid energy in favor of clean energy.

**Table 11:** Mechanism analysis results.

	Improvement of energy-efficient appliances		Increased awareness of environmental protection	
	OLS	IV	OLS	IV
*ln n oninc*	0.020^***^ (0.007)	0.741 ^***^ (0.184)	0.022^***^ (0.004)	0.562^***^ (0.199)
Householder characteristics	√	√	√	√
Family characteristics	√	√	√	√
Village fixed effect	√	√	√	√
Year fixed effect	√	√	√	√
Intercept	6.274^***^ (0.203)	−3.353^**^ (1.584)	6.295^***^ (0.138)	1.408 (1.717)
Durbin–Wu–Hausman *p*-value		0.000		0.001
Sargan test *p*-value		0.174		0.162
Observations	6,349	6,349	6,349	6,349

() is the clustering robust standard error at the village level, ^*^statistical significance at 10% level, ^**^statistical significance at 5% level, ^***^statistical significance at 1% level.

#### The impact of non-farming income on improvement of environmental awareness

To investigate the mechanism whereby non-farming income affects rural households’ environmental awareness, we use the answers—from 1 (totally unnecessary) to 10 (absolutely necessary)—of household members to the question “*Do you think the environmental pollution problem in China needs to be addressed vigorously.”* Considering that family members will influence each other; the average environmental awareness of all adult family members is adopted as the measure of the overall environmental awareness of rural households. The results in Table 11 show that an increase in non-farming income improves the environmental awareness of households and thus has an impact on energy choice. By traveling to urban areas to engage in non-farming work, rural households can effectively promote the transmission of environmental protection ideas, thereby enhancing their environmental awareness (Zhou and Yang, 2009).

## Conclusions and policy implications

Using CFPS data on household non-farming income and energy use, this study first establishes empirical evidence that there is a synergistic effect between non-farming income and energy improvement. On this basis, OLS and IV estimation methods are used to investigate the causal effect of non-farming income on household energy choice, indicating that an increase in rural households’ non-farming income would have a significant negative impact on the use of traditional non-clean energy (firewood, coal), and a significant positive impact on the use of cleaner energy (bottled gas, natural gas, electricity). The conclusion was robust and follows the expectation of the energy ladder theory. In addition, during the COVID-19 pandemic, the government implemented a “dynamic zero” epidemic prevention policy, which also might influence people’s energy choices (Jia et al., 2022). Future research is thus recommended to investigate whether the rural household energy choices will be further changed during COVID-19.

Furthermore, by distinguishing the income level of villages and their distance from the center of the county seat, our heterogeneity analysis shows that the non-farming income of households in high-income villages has a stronger effect on the energy transition than in low-income areas. Moreover, the increasing effect of non-farming income on the use of natural gas is stronger in suburban areas, while the decreasing effect of non-farming income on households’ use of firewood is stronger in the exurbs. We also find that an increase in non-farming income mainly affects the improvement of household energy use structure through the improvement of household energy-efficient appliances and the enhancement of environmental awareness.

The conclusions of this study have obvious policy implications. First, to achieve the goal of China’s energy transformation and upgrading, and in addition to a series of emission reduction policies, the government should formulate appropriate employment support policies, to increase the opportunities for rural households and encourage them to venture out and engage in non-farming employment, thereby improving their non-farming income. For example, Gansu and Hebei developed the wind energy and wind power stations to change people’s energy choices. Second, the government should focus on the development of low-income and exurb village residents through rural subsidies and industrial support policies, narrow the income gap among village residents, and highlight the poverty reduction effect of the energy transition. Moreover, the government should enhance the governance of the exurb villagers and strengthen the implementation of policies in these areas. Various provinces introduced proposals, such as Fujian, Jiangsu, Henan, Shandong and Ningxia provinces have developed incentive-based policies to advance the extension and application of solar water heaters (Zeng et al., 2019). Third, considering that the improvement and upgrading of household energy-efficient appliances and the awareness of environmental protection have a positive effect on the energy transition, the government should introduce subsidization policies for new coal-fired and gas stoves, promote the transition to energy-efficient appliances, and strengthen their publicity around environmental protection.

## Data availability statement

Publicly available datasets were analyzed in this study. This data can be found at: the China Family Panel Studies (https://opendata.pku.edu.cn/dataverse/CFPS?language=en).

## Author contributions

All authors listed have made a substantial, direct, and intellectual contribution to the work and approved it for publication.

## Funding

This research was supported by a grant from the National Social Science Foundation of China (No. 21BTJ020).

## Conflict of interest

The authors declare that the research was conducted in the absence of any commercial or financial relationships that could be construed as a potential conflict of interest.

## Publisher’s note

All claims expressed in this article are solely those of the authors and do not necessarily represent those of their affiliated organizations, or those of the publisher, the editors and the reviewers. Any product that may be evaluated in this article, or claim that may be made by its manufacturer, is not guaranteed or endorsed by the publisher.

## Supplementary material

The Supplementary material for this article can be found online at: https://www.frontiersin.org/articles/10.3389/fpsyg.2022.1044362/full#supplementary-material
