# Supplementary material for: The impacts of non-farming income on rural household energy choices: Empirical evidence from China
Source: Front Psychol. 2022 Oct 21;13:1044362. doi: 10.3389/fpsyg.2022.1044362 (PMC9635446; doi:10.3389/fpsyg.2022.1044362)
Supplement: Supplementary file 1 [file Data_Sheet_1.docx]

**Supplementary**

**Table A1.** Complete results of baseline regression

|  | **Column one** | **Column two** | **Column three** |
| --- | --- | --- | --- |
| **Panel A Firewood (**$firewood$**)** | | | |
| Non-farming income | -0.014^***^(0.001) | -0.007^***^(0.001) | -0.009^***^(0.0008) |
| The gender of householders |  | -0.087^***^(0.007) | -0.086^***^(0.007) |
| The age of householder |  | 0.001^*^(0.0003) | 0.001^***^(0.0003) |
| The education level of householders |  | -0.031^***^(0.001) | -0.031^***^(0.001) |
| The health of householders |  | -0.014^***^(0.003) | -0.013^***^(0.003) |
| Young population in the family |  |  | 0.054^***^(0.021) |
| Old population the family |  |  | -0.051^***^(0.015) |
| Village fixed effect | YES | YES | YES |
| Year fixed effect | YES | YES | YES |
| Intercept | 0.085 ^***^(0.017) | 0.681^***^(0.061) | 0.602^***^(0.064) |
| $R^{2}$ | 0.013 | 0.051 | 0.051 |
| Observations | 19,451 | 19,451 | 19,451 |
| **Panel B Coal (**$coal$**)** | | | |
| Non-farming income | -0.001^***^(0.0004) | -0.001^***^(0.0004) | -0.0008^*^(0.0004) |
| The gender of householders |  | -0.005(0.004) | -0.006(0.004) |
| The age of householder |  | 0.0002(0.0001) | 0.0001(0.0002) |
| The education level of householders |  | -4.77e-05(0.0006) | 0.0001(0.0006) |
| The health of householders |  | 0.003^**^(0.001) | 0.003^**^(0.001) |
| Young population in the family |  |  | 0.030^***^(0.011) |
| Old population the family |  |  | 0.021^***^(0.008) |
| Village fixed effect | YES | YES | YES |
| Year fixed effect | YES | YES | YES |
| Intercept | -1.458***  (0.025) | -1.599^***^(0.092) | -1.641^***^(0.097) |
| $R^{2}$ | 0.001 | 0.002 | 0.004 |
| Observations | 19,451 | 19,451 | 19,451 |
| **Panel C Bottled gas (**$botgas$**)** | | | |
| Non-farming income | 0.010^***^(0.0007) | 0.008^***^(0.0007) | 0.008^***^(0.0007) |
| The gender of householders |  | 0.071^***^(0.006) | 0.071^***^(0.006) |
| The age of householder |  | 0.0006^***^(0.0002) | 0.0004(0.0003) |
| The education level of householders |  | 0.015^***^(0.001) | 0.015^***^(0.001) |
| The health of householders |  | 0.004(0.002) | 0.004(0.002) |
| Young population in the family |  |  | -0.013(0.018) |
| Old population the family |  |  | 0.024^*^(0.014) |
| Village fixed effect | YES | YES | YES |
| Year fixed effect | YES | YES | YES |
| Intercept | -0.940^***^(0.020) | -1.489^***^(0.068) | -1.459^***^(0.070) |
| $R^{2}$ | 0.011 | 0.026 | 0.026 |
| Observations | 19,451 | 19,451 | 19,451 |
| **Panel D Natural gas (**$natgas$**)** | | | |
| Non-farming income | 0.003^***^(0.0003) | 0.002^***^(0.0003) | 0.002^***^(0.0003) |
| The gender of householders |  | 0.009^***^(0.003) | 0.009^***^(0.003) |
| The age of householder |  | -0.0004^***^(0.0001) | -0.0005^***^(0.0001) |
| The education level of householders |  | 0.005^***^(0.0004) | 0.004^***^(0.0004) |
| The health of householders |  | 0.002^*^(0.001) | 0.002^*^(0.001) |
| Young population in the family |  |  | -0.036^***^(0.008) |
| Old population the family |  |  | 0.0002(0.006) |
| Village fixed effect | YES | YES | YES |
| Year fixed effect | YES | YES | YES |
| Intercept | -2.183^***^(0.042) | -2.414^***^(0.127) | -2.256^***^(0.132) |
| $R^{2}$ | 0.016 | 0.064 | 0.068 |
| Observations | 19,451 | 19,451 | 19451 |
| **Panel E Electricity (**$electric$**)** | | | |
| Non-farming income | 0.002^***^(0.0006) | 0.001(0.001) | 0.001(0.001) |
| The gender of householders |  | 0.015^**^(0.006) | 0.016^***^(0.006) |
| The age of householders |  | 0.0003(0.0002) | 0.0005^**^(0.0002) |
| The education level of householders |  | 0.008^***^(0.0009) | 0.008^***^(0.001) |
| The health of householders |  | 0.006^***^(0.002) | 0.006^***^(0.002) |
| Young population in the family |  |  | 0.0061(0.017) |
| Old population the family |  |  | -0.025^*^(0.013) |
| Village fixed effect | YES | YES | YES |
| Year fixed effect | YES | YES | YES |
| Intercept | -0.862^***^(0.019) | -1.162^***^(0.068) | -1.187^***^(0.071) |
| $R^{2}$ | 0.0004 | 0.004 | 0.005 |
| Observations | 19,451 | 19,451 | 19,451 |

Notes: () is the clustering robust standard error at the village level, *statistical signiﬁcance at 10% level, **statistical signiﬁcance at 5% level, ***statistical signiﬁcance at 1% level.

**Table A2.** Regression results of instrumental variables

|  | **Column one** | **Column two** | **Column three** |
| --- | --- | --- | --- |
| **Firewood (**$\boldsymbol{firewood}$**)** | | | |
| Non-farming income | -0.041^***^(0.003) | -0.033^***^(0.004) | -0.037^***^(0.005) |
| The gender of householders |  | -0.073^***^(0.008) | -0.071^***^(0.008) |
| The age of householders |  | -0.001^***^(0.0004) | 3.37e-05(0.0004) |
| The education level of householders |  | -0.027^***^(0.001) | -0.026^***^(0.001) |
| The health of householders |  | -0.014^***^(0.003) | -0.013^***^(0.003) |
| Young population in the family |  |  | 0.036^*^(0.021) |
| Old population the family |  |  | -0.175^***^(0.027) |
| Intercept | 0.729***(0.024) | 1.002***(0.049) | 0.964***(0.048) |
| Durbin-Wu-Hausman P-Value | 0.000 | 0.000 | 0.000 |
| Sargan test P-Value | 0.022 | 0.121 | 0.161 |
| Observations | 19,445 | 19,445 | 19,445 |
| **Coal (**$\boldsymbol{coal}$**)** | | | |
| Non-farming income | -0.002(0.002) | -0.003(0.002) | -0.002(0.002) |
| The gender of householders |  | -0.004(0.004) | -0.005(0.004) |
| The age of householders |  | 8.05e-05(0.0002) | 5.24e-05(0.0002) |
| The education level of householders |  | 0.0002(0.0006) | 0.0004(0.001) |
| The health of householders |  | 0.003^**^(0.001) | 0.003^**^(0.001) |
| Young population in the family |  |  | 0.029^***^(0.011) |
| Old population the family |  |  | 0.015(0.013) |
| Intercept | 0.081^***^ (0.012) | 0.069^***^(0.024) | 0.062^***^(0.024) |
| Durbin-Wu-Hausman P-Value | 0.001 | 0.001 | 0.001 |
| Sargan test P-Value | 0.448 | 0.476 | 0.526 |
| Observations | 19,445 | 19,445 | 19,445 |
| **Bottled gas (**$\boldsymbol{botgas}$**)** | | | |
| Non-farming income | 0.012^***^(0.003) | 0.007^**^(0.004) | 0.009^**^(0.004) |
| The gender of householders |  | 0.072^***^(0.007) | 0.071^***^(0.007) |
| The age of householders |  | 0.0005(0.0004) | 0.0003(0.0003) |
| The education level of householders |  | 0.016^***^(0.001) | 0.015^***^(0.001) |
| The health of householders |  | 0.004(0.002) | 0.003(0.002) |
| Young population in the family |  |  | -0.014(0.018) |
| Old population the family |  |  | 0.027(0.0232) |
| Intercept | 0.157^***^(0.021) | 0.017(0.042) | 0.017(0.041) |
| Durbin-Wu-Hausman P-Value | 0.000 | 0.001 | 0.001 |
| Sargan test P-Value | 0.420 | 0.715 | 0.867 |
| Observations | 19,445 | 19,445 | 19,445 |
| **Natural gas (**$\boldsymbol{natgas}$**)** | | | |
| Non-farming income | 0.016^***^(0.001) | 0.016^***^(0.002) | 0.018^***^(0.002) |
| The gender of householders |  | 0.004(0.003) | 0.002(0.003) |
| The age of householders |  | 0.001^***^(0.0002) | -2.01e-05(0.0001) |
| The education level of householders |  | 0.003^***^(0.0005) | 0.003^***^(0.0005) |
| The health of householders |  | 0.002^*^(0.001) | 0.002(0.001) |
| Young population in the family |  |  | -0.031^***^(0.008) |
| Old population the family |  |  | 0.084^***^(0.010) |
| Intercept | -0.084^***^(0.009) | -0.157^***^(0.018) | -0.132^***^(0.018) |
| Durbin-Wu-Hausman P-Value | 0.000 | 0.002 | 0.000 |
| Sargan test P-Value | 0.129 | 0.664 | 0.669 |
| Observations | 19,445 | 19,445 | 19,445 |
| **Electricity (**$\boldsymbol{electric}$**)** | | | |
| Non-farming income | 0.008^***^(0.003) | 0.007^**^(0.004) | 0.008^**^(0.004) |
| The gender of householders |  | 0.012^**^(0.006) | 0.012^*^(0.006) |
| The age of householders |  | 0.0008^**^(0.0004) | 0.0008^***^(0.0003) |
| The education level of householders |  | 0.007^***^(0.001) | 0.007^***^(0.001) |
| The health of householders |  | 0.006^***^(0.002) | 0.006^***^(0.002) |
| Young population in the family |  |  | 0.010(0.017) |
| Old population the family |  |  | 0.008(0.022) |
| Intercept | 0.148^***^(0.020) | 0.043(0.040) | 0.039(0.039) |
| Durbin-Wu-Hausman P-Value | 0.012 | 0.057 | 0.059 |
| Sargan test P-Value | 0.899 | 0.857 | 0.862 |
| Observations | 19,445 | 19,445 | 19,445 |

Notes: () is the clustering robust standard error at the village level, *statistical signiﬁcance at 10% level, **statistical signiﬁcance at 5% level, ***statistical signiﬁcance at 1% level.
